# Supplementary material for: Merkel Cell Polyomavirus T Antigens Induce Merkel Cell-Like Differentiation in GLI1-Expressing Epithelial Cells
Source: Cancers (Basel). 2020 Jul 21;12(7):1989. doi: 10.3390/cancers12071989 (PMC7409360; doi:10.3390/cancers12071989)
Supplement: Supplementary file 1 [file cancers-12-01989-s001.pdf]

*Supplementary Matreials*

# MCPyV T Antigens Induce Merkel Cell-Like Differentiation in GLI1-Expressing Epithelial Cells

**Thibault Kervarrec [1,2,3,*], Mahtab Samimi [2,4], Sonja Hesbacher [3], Patricia Berthon [2], Marion Wobser [3], Aurélie Sallot [5], Bhavishya Sarma [3], Sophie Schweinitzer [3], Théo Gandon [2], Christophe Destrieux [6], Côme Pasqualin [7], Serge Guyétant [1,2], Antoine Touzé [2], Roland Houben [3,†] and David Schrama [3,†]**

**Table S1.** Antibodies used for immunohistochemistry.

| Antigen | Clone | Company | Dilution |
|---|---|---|---|
| Large T | CM2B4 | Santa Cruz | 1/50 |
| KRT8 | M20 | Santa Cruz | 1/50 |
| KRT14 | SP53 | Dako | pre-diluted |
| KRT17 | Ks17.E3 | Santa Cruz | 1/200 |
| KRT18 | DC-10 | Santa Cruz | 1/100 |
| KRT20 | Ks20.8 | Dako | 1/100 |
| GLI1 | C68H3 | Ozyme | 1/200 |
| Neurofilament | 2F11 | Dako | pre-diluted |
| SOX2 | EPR3131 | Abcam | 1/50 |
| SOX9 | AB5535 | Merck | 1/1000 |

KRT: keratin; GLI1: GLI family zinc finger 1, SOX2: SRY-box 2, SOX9: SRY-box 9.

**Table S2.** Primers sequences.

| Gene | Sequence |
|---|---|
| ATOH1 fw | ACTTGCCTCATCCGAGTCAC |
| ATOH1 rv | GCAGGAGGAAAACAGCAAAA |
| GLi1 fw | CCTTCAGCAATGCCAGTG |
| GLi1 rv | GCTTACATACATACGGCTTCTC |
| KRT8 fw | TGGAGCAGCAGAACAAGATG |
| KRT8 Rv | CCGCCTAAGGTTGTTGATGT |
| KRT14 fw | AGAGGACGCCCACCTTTC |
| KRT14 rv | TTAGTTCTTGGTGCGCAG |
| KRT17 fw | CCCACTTGGTGGCCTATAAA |
| KRT17 rv | GTCATCAGGCAAGGAAGCAT |
| KRT18 fw | TAGATGCCCCCAAATCTCAG |
| KRT18 rv | CACTGTGGTGCTCTCCTCAA |
| KRT20 fw | GGACGACACCCAGCGTTTAT |
| KRT20 rv | CGCTCCCATAGTTCACCGTG |
| RPLP0 fw | CCATCAGCACCACAGCCTTC |
| RPLP0 rv | GGCGACCTGGAAGTCCAACT |
| SOX2 fw | GCTTAGCCTCGTCGATGAAC |
| SOX2 rv | AACCCCAAGATGCACAACTC |
| SOX9 fw | GGAGATGAAATCTGTTCTGGGAATG |
| SOX9 rv | TTGAAGGTTAACTGCTGGTGTTCTG |

ATOH1: Atonal homolog 1; KRT: keratin; GLI1: GLI family zinc finger 1; RPLP0: ribosomal protein lateral stalk subunit P0; SOX2: SRY-box 2, SOX9: SRY-box 9.

**Table S3.** Merkel cells distribution subdivided by anatomic site.

| Subject | Anatomic Site | MCs Counts (50 Sections) | Estimated MC Density (nb/mm$^2$) | MCs Hotspot Count | Estimated MCs Hotspot Density (nb/mm$^2$) |
|---|---|---|---|---|---|
| N°1 | Scalp | 133 | 90 | 14 | 9 |
| | Face | 40 | 27 | 2 | 1 |
| | Trunk | 36 | 53 | 2 | 1 |
| | Finger | 146 | 99 | 11 | 7 |
| | Legs | 54 | 79 | 4 | 2 |
| N°2 | Scalp | 94 | 64 | 10 | 7 |
| | Face | 62 | 91 | 1 | 1 |
| | Trunk | 7 | 5 | 0 | 0 |
| | Finger | 272 | 184 | 23 | 15 |
| | Legs | 12 | 8 | 0 | 0 |
| N°3 | Scalp | 39 | 26 | 3 | 2 |
| | Face | 120 | 81 | 9 | 6 |
| | Trunk | 11 | 7 | 1 | 1 |
| | Finger | 42 | 28 | 3 | 2 |
| | Legs | 33 | 22 | 4 | 3 |

MC: Merkel cell, nb: number.

**Table S4.** Expression of the MC progenitor and MC markers in the trichoblastoma and MCC tumors.

| Markers expression in Tumors | Trichoblastoma | | MCC* | |
|---|---|---|---|---|
| | Germinative Cells | Merkel Cells | MCPyV-Positive | MCPyV-Negative |
| **MC progenitor markers** | | | | |
| GLI1** | 7/8 | NA | 16/66 | 10/21 |
| SOX9** | 8/8 | NA | 7/69 | 17/21 |
| KRT17 | 8/8 | NA | 0 | 0 |
| **MC markers** | | | | |
| SOX2 | 0 | 5/6 | 75/75 | 19/21 |
| KRT20 | 0 | 8/8 | 68/74 | 19/21 |

NA: non-applicable (since diffuse expression was observed, specific MC expression could not be determined), *: results are expressed in number of interpretable cases (for MCC samples only samples with available MCPyV status and interpretable staining were included); ** only cases in which nuclear expression was seen are regarded as positive.

**Table S5.** Expression of GLI1 and SOX9 in Merkel cell polyomavirus positive and negative Merkel cell carcinomas.

| | MCPyV(−) MCC | MCPyV(+) MCC | Unknown Status | *p** |
|---|---|---|---|---|
| **GLI1** | | | | |
| Negative (*n* = 60) | 10 (48%) | 50 (76%) | 0 | 0.028 |
| Positive (*N* = 29) | 11 (52%) | 16 (24%) | 2 | |
| Unavailable data | 1 | 12 | 1 | |
| **SOX9** | | | | |
| Negative | 1 (5%) | 6 (9%) | 0 | $<1\times10^{-9}$ |
| Dot-like | 3 (14%) | 56 (81%) | 2 | |
| Patchy | 17 (81%) | 7 (10%) | 0 | |
| Unavailable data | 1 | 10 | 1 | |

GLI1: GLI family zinc finger 1, MCC: Merkel cell carcinoma, MCPyV: Merkel cell Polyomavirus, SOX9: SRY-box 9, * variables were compared by Fisher's tests, and *p* values < 0.05 were considered as significant.

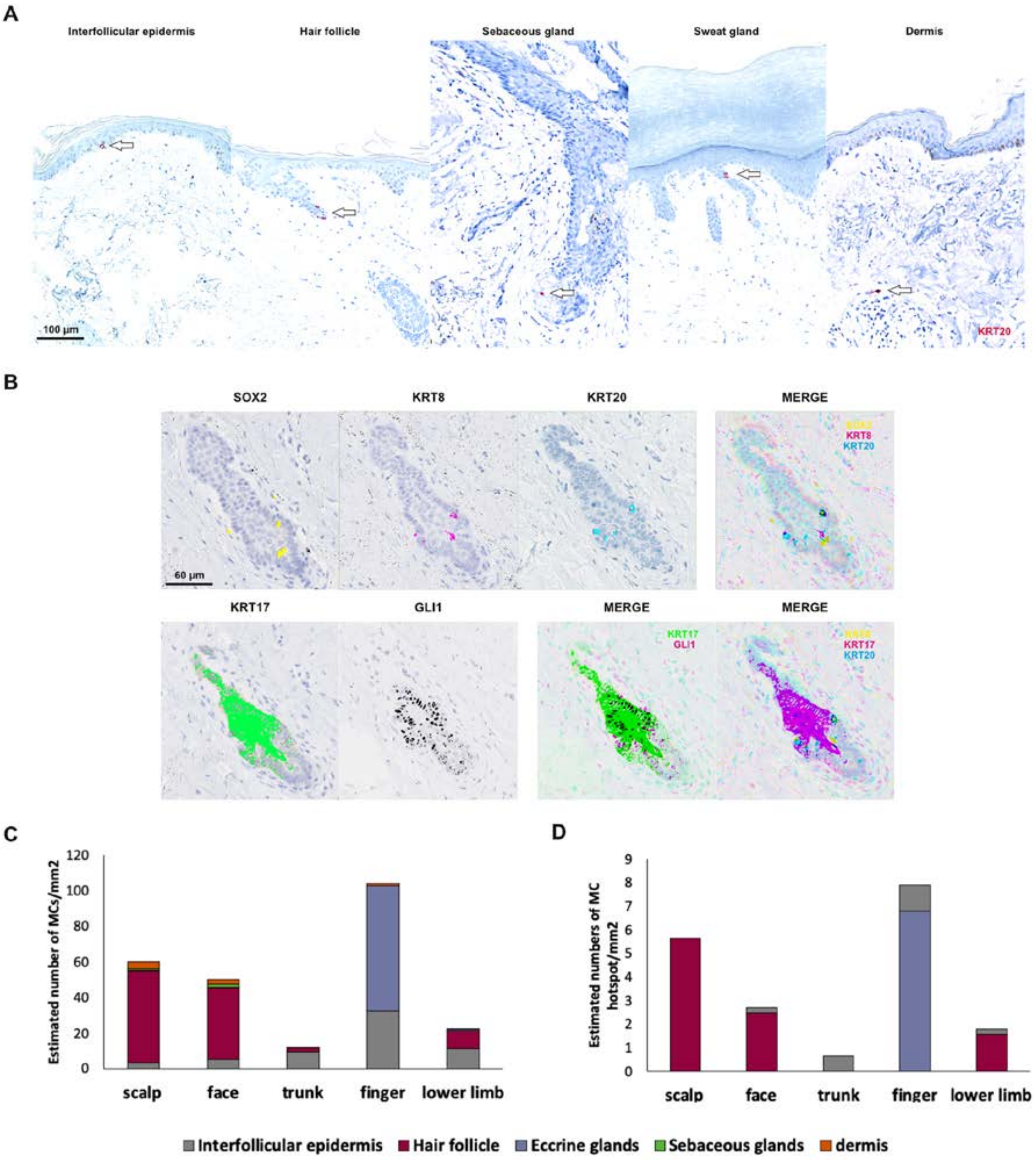


**Figure S1. Further characterization of Merkel cells and related progenitors in human: A:** Merkel cell **(**MC) locations in human. KRT20-expressing MCs were observed in the interfollicular epidermis, in appendages structures and in few cases in the dermis (bar = 100 μm). MCs were mostly located in hair follicles (43% of the observed MCs). There, MCs were detected in infundibulum (80%) or in the isthmus part (20%). The proximal part of the eccrine sweat gland duct was also a MCs niche mostly in acral skin. Of note, in contrast to the previous reports, some MCs were also observed in the papillary dermis, frequently in close proximity to the hair follicle. These findings suggest that MCs might experience a epithelia-mesenchymal transition process. **B:** MCs markers in human: merged analyses confirmed co-localisation of the MCs markers (KRT8, KRT20 and SOX2). Moreover, co-location of GLI1 and KRT17 expression could also be detected. Of note, expression of the MC progenitors' markers GLI1 and KRT17 seems to be reduced in MCs. **C:** MCs density according to the anatomic sites and microscopic location. **D:** MC hotspot densities per location and anatomic sites.

**A**

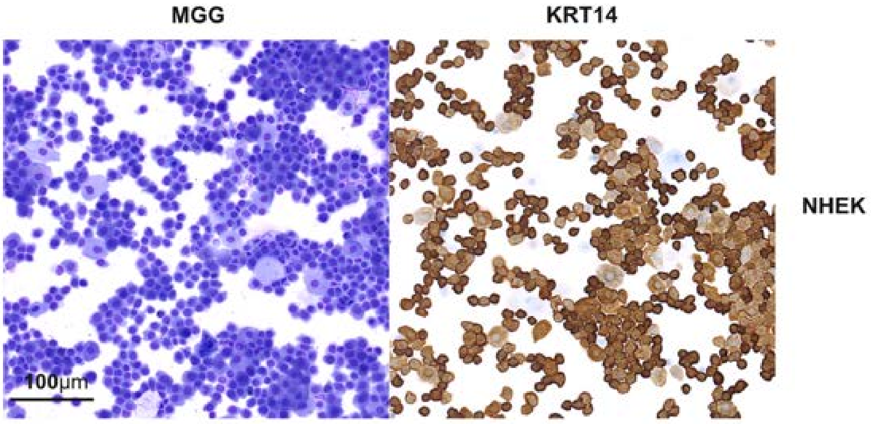


**B**

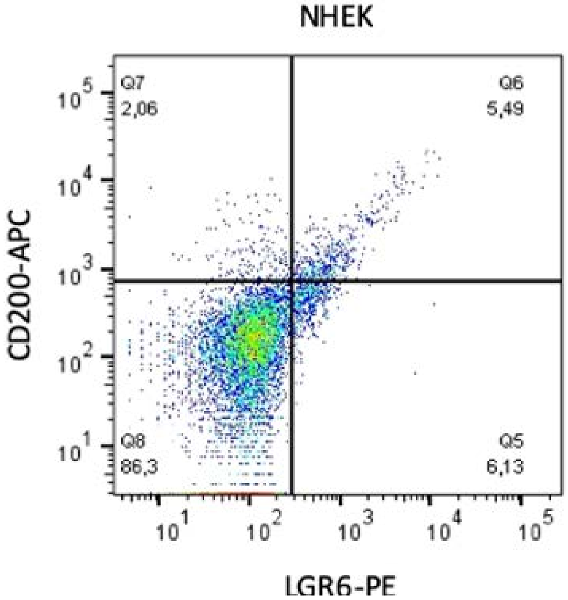


**Figure S2. Characterization of NHEK cells. A**: After isolation from surgical piece, morphology of normal human epidermal keratinocytes (NHEK) was assessed (May Grunwald Giemsa staining), and expression of KRT14, a basal keratinocyte marker, was confirmed in this population. **B**. Expression of the stem cell/progenitor markers lgr6 and CD200 by flow cytometry in NHEK.

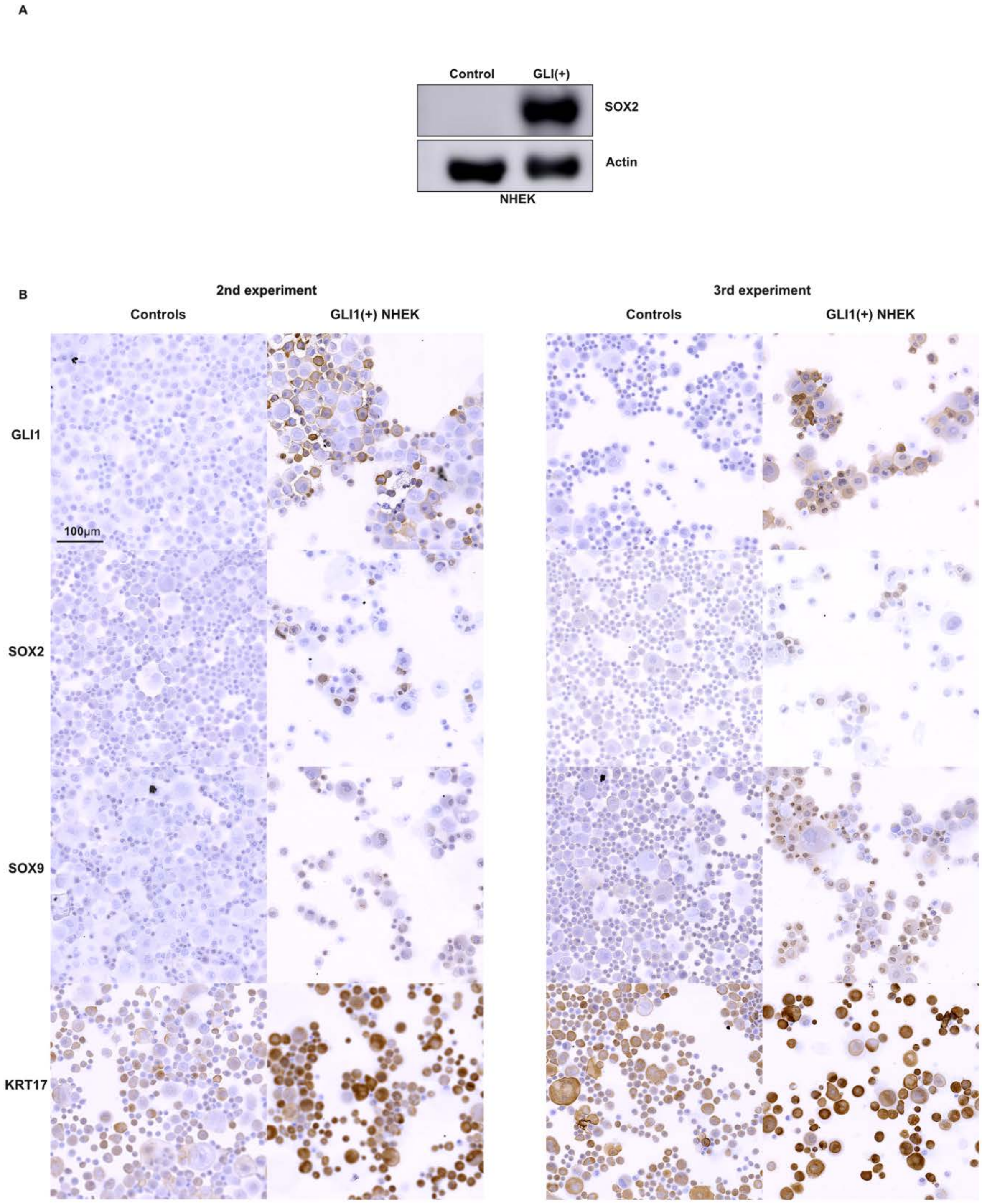


**Figure S3.** Repetitive experiments demonstrating ectopic GLI1 expression-mediated induction of several MC lineage markers in NHEK. **A:** Detection of SOX2 expression by immunoblot in GLI-transduced NHEK and controls (expected size: 35KDa). **B**: Immunohistochemical assessment of GLI1, SOX2, SOX9 and KRT17 expression levels in GLI1-expressing NHEK and control (two additional independent experiments).

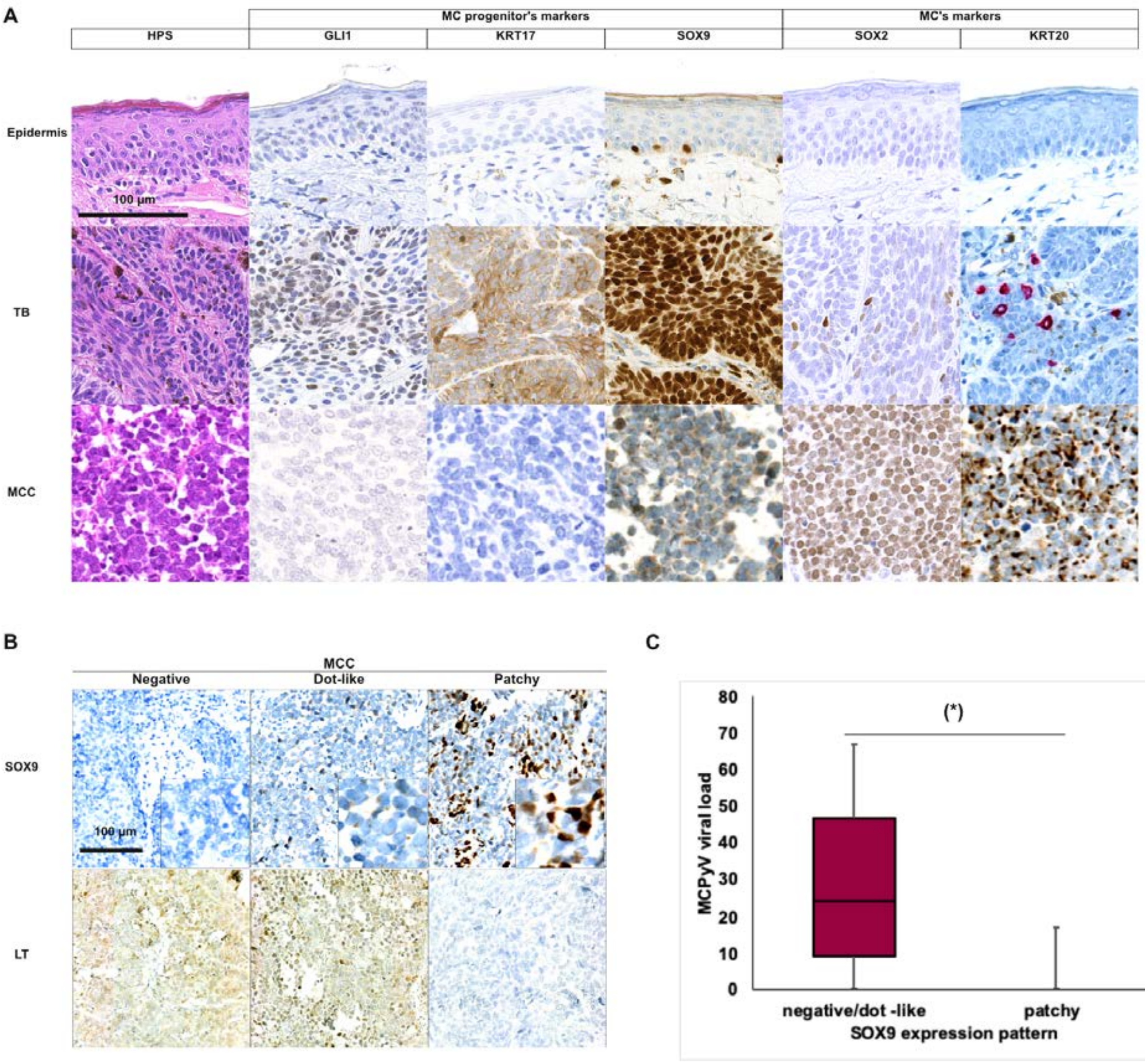


**Figure S4. Expression of Merkel cell progenitor markers in tumors.** **A**: microscopic and immunophenotypic features of Trichoblastoma (TB) and Merkel cell carcinoma (MCC) tumors: Morphology (hematein-phloxin-saffran staining), expression of the progenitor markers GLI1, KRT17 and SOX9 as well as the Merkel cell markers SOX2 and KRT20 in TB and MCC are depicted. Surrounding epidermis was used as control (bar=100 µm). **B:** Expression pattern of SOX9 in Merkel cell polyomavirus (MCPyV)-positive and -negative MCC (bar=100 -µm). While nuclear positivity for GLI1 was always homogenous, a patchy pattern consisting of some tumor cells expressing SOX9 cytoplasmic or nuclear suggests that in such cases only a subpopulation of tumor cells harbored the active form of SOX9. Of note, most of the cases without SOX9 nuclear positivity were characterized by a hitherto undescribed paranuclear dot like SOX9 staining (64%) suggesting a potential sequestration of this factor in the cytoplasm or the Golgi apparatus. Since it has been previously postulated that MCPyV-negative cases contain a subpopulation of pluripotent progenitor cells explaining why divergent differentiation only occurs in this MCC subset [72,73], we investigated expression of GLI1 and SOX9 according to the MCPyV status. In line with this hypothesis, SOX9 nuclear positivity (81 versus 10%) was more frequently observed in MCPyV-negative MCC ($p<0.03$ and $p<10^{-9}$, respectively) (**supplementary Table 2**). **C:** MCPyV viral load of the MCC cases subdivided by SOX9 expression pattern ($p<0.05$).

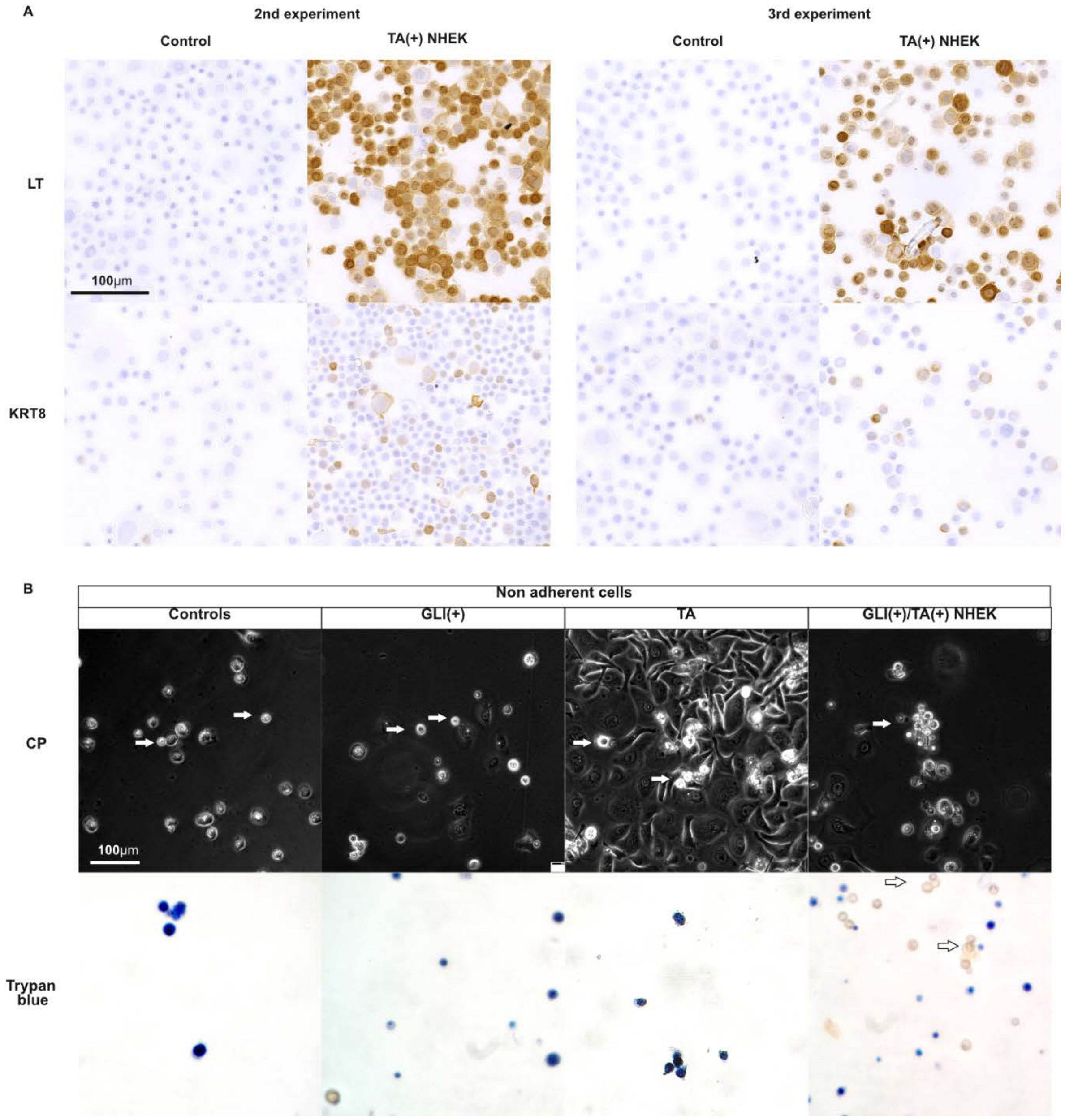


**Figure S5.** Continued.

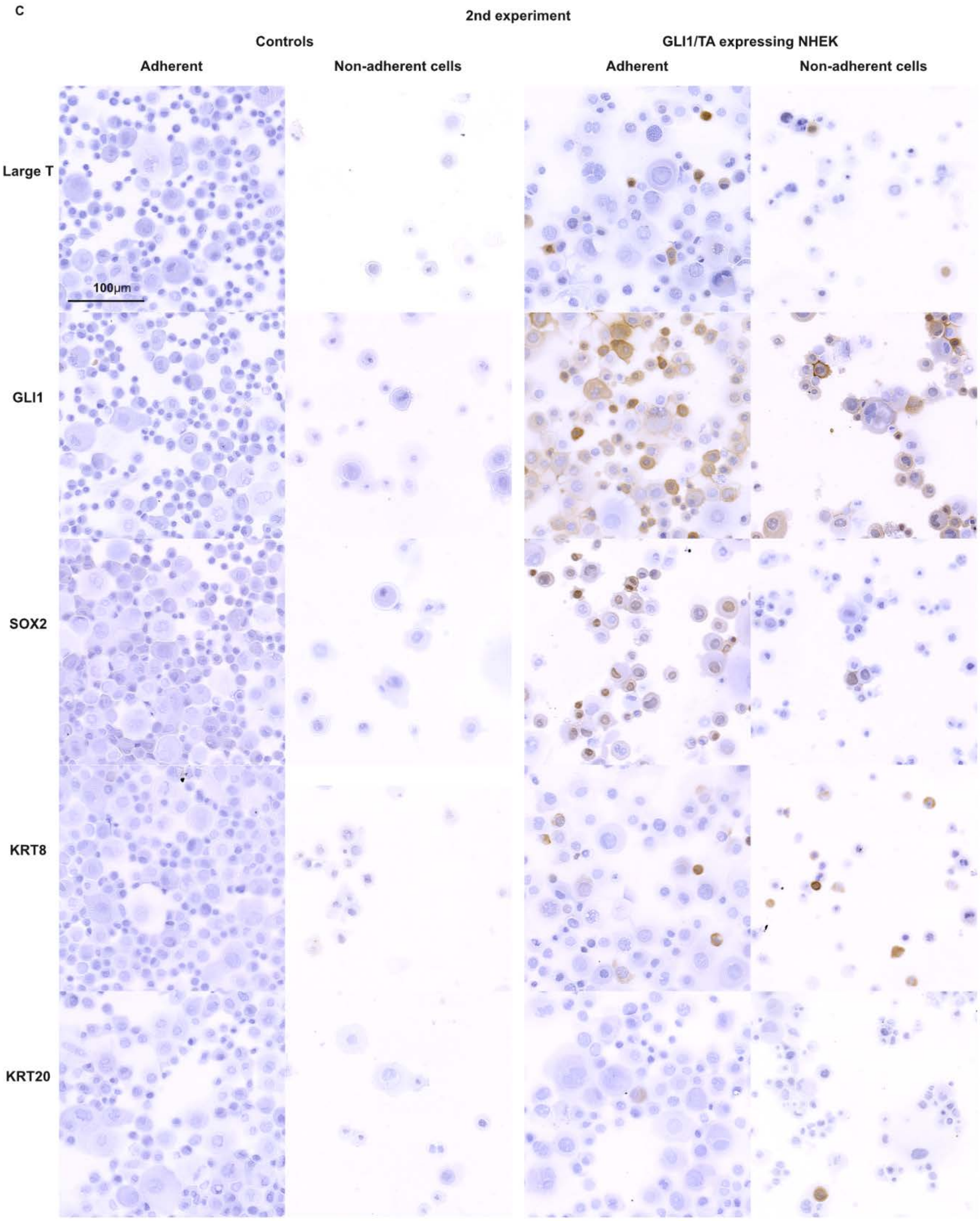


**Figure 5.** Continued.

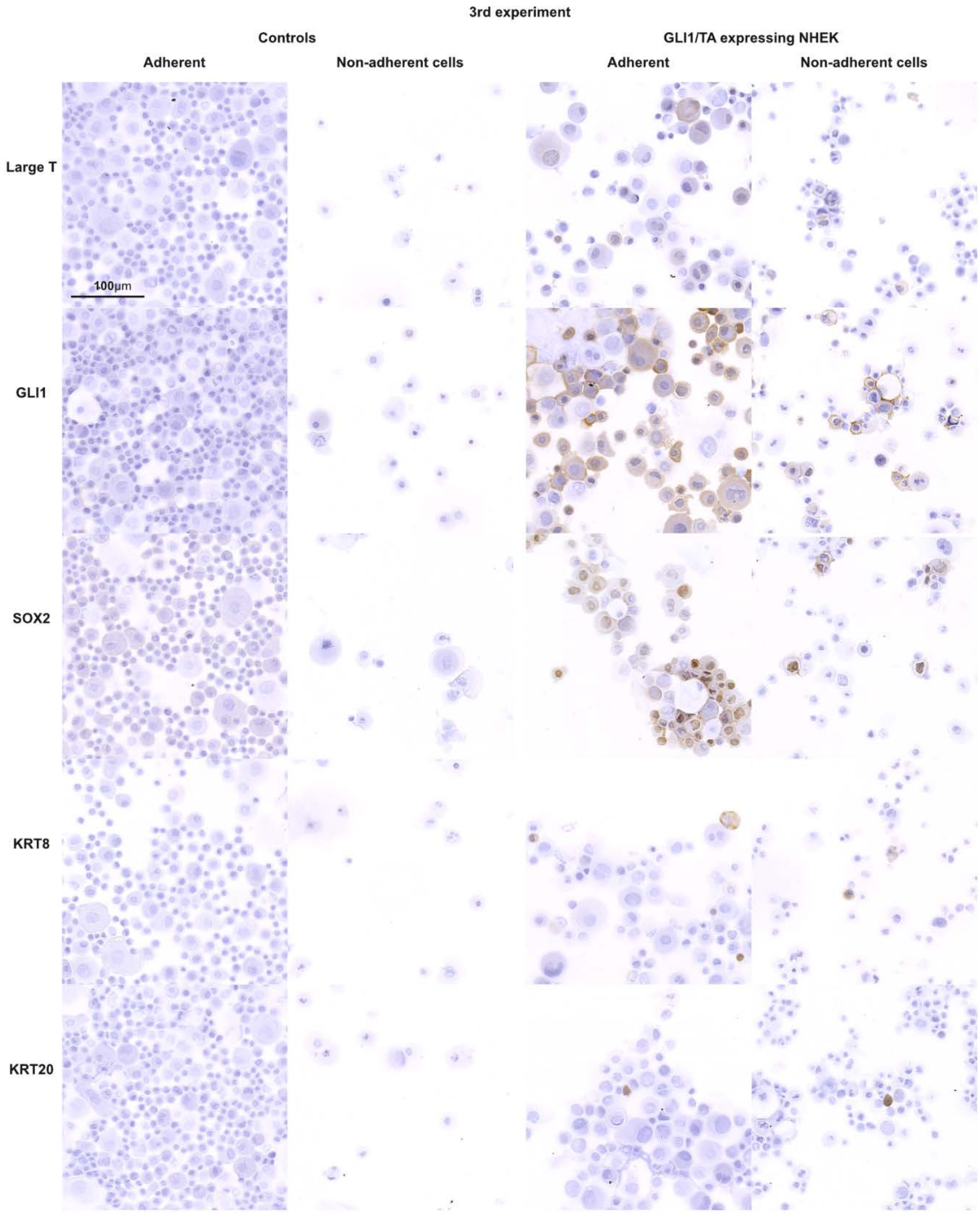


**Figure S5.** Continued.

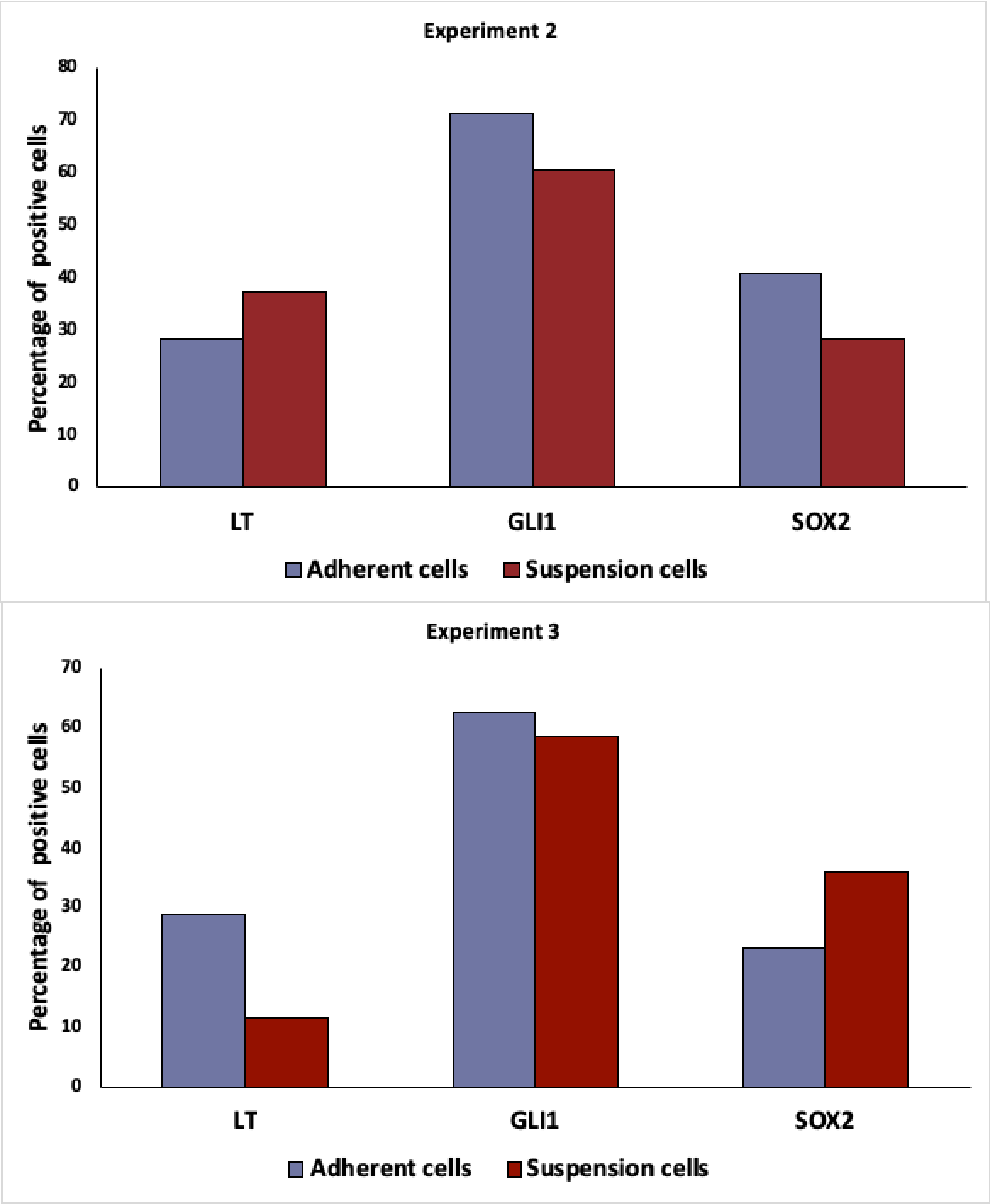


D

**Figure S5.** Repetitive experiments demonstrating TA and GLI1/ TA-mediated induction of MC lineage marker in NHEK. **A**: Immunohistochemical assessment of Large T (LT) and keratin 8 (KRT8) in T antigen (TA)-expressing NHEK and controls in a second and a third experiment. **B**: Assessment of the morphology and viability of non-adherent cells in TA(+), GLI1(+) GLI1(+)/TA(+) NHEK and controls: floating but still living cells were only observed in the GLI1/TA co-expressing cells after antibiotic selection. **C**: Immunohistochemical assessment of Large T, GLI1, SOX2, KRT8 and KRT20 expression levels in GLI1/TA-coexpressing NHEK and controls (two additional independent experiments). After transduction and antibiotic selection, adherent and non-adherent cells were analyzed independently. This analysis demonstrated positivity of SOX2, keratin (KRT) 8 and 20 in both GLI(+)/T Antigens (TA)(+) adherent and non adherent Normal human epidermal keratinocytes (NHEK). **D**: Quantification of LT, GLI1 and SOX2 non expressing and expressing cells in adherent and non-adherent GLI1/TA-coexpressing NHEKs, cells counts was preformed on 10 consecutive fields at high magnification (x40).

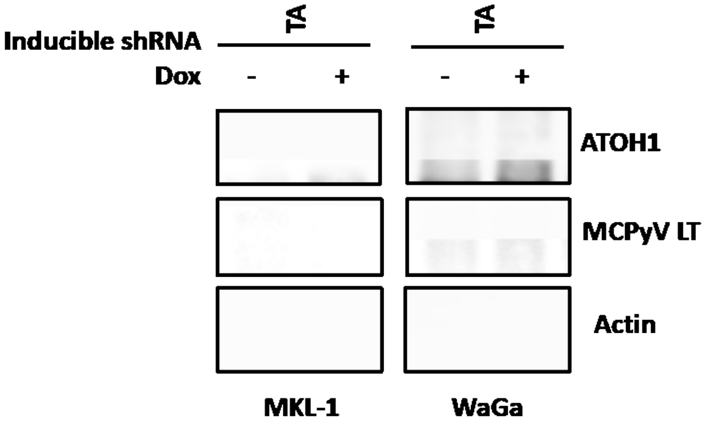


**Figure S6. Impact of TA knock-down on ATOH1 protein level in the MCC cell lines MKL-1 and WaGa. The** MCPyV positive cell lines were transduced with a Dox-inducible TA.shRNA. tet vector system and ATOH1 endogenous level was evaluated after 4 days of Doxycyclin (Dox) exposure.

This image cannot currently be displayed.

**Figure S7. ATOH1 stabilization by mutation of phosphosites or delta Mur1 containing LT. A:** Evaluation of ATOH1 mutated protein half-life in comparison to the wild type form. Phosphorylation of the serine residues S331, S337, S341 of the ATOH1 protein are expected to control protein degradation. Therefore, these serine residues were changed to alanine in three independent constructs in order to remove the phosphorylation site. Half-lifes of the obtained constructs were evaluated with cycloheximide chase experiments. An increased half-life was observed for S337 and S341 ATOH1 mutants. **B:** Impact of T antigens (TA) on protein level of ATOH1-HA wild type and single mutants. **C:** Evaluation of the individual effect of truncated Large T (LT) or small T (sT) on ATOH1-HA protein level assessed by immunoblot in U2OS cells. ATOH1-HA protein level was assessed by immunoblot after transfection of an increased amounts of LT or sT DNA. Such analysis identified LT as the main contributor of ATOH1-HA stabilization. **D:** Evaluation of the ability of mutated forms of truncated Large T antigen with inactivated functional sites (S220A: mutant lacking LT phosphosite, D44N:

mutant with inactivated HSP70 binding site, E216K: mutant with inactivated RB1 binding site, ΔMUR1: mutant deleted for the Merkel unique 1 region (MUR1)) to stabilize ATOH1-HA. Quantification of ATOH1-HA expression levels was performed using ImageJ software. Of note, since the antibody used for LT detection (clone: CM2B4) binds to a peptide from MUR1, no signal was obtained with the LT-ΔMUR1 mutant. **E-F:** further confirmation of the lack of ATOH1-HA stabilization by LT-ΔMUR1 mutant. **E:** ATOH1-HA construct was transfected in absence or presence of increased amounts of TA, a V5-tagged LT wild type and V5-tagged LT-ΔMUR1 DNA. Evaluation of ATOH1 by immunoblot revealed an accumulation of ATOH1-HA protein in presence of wild type truncated LT while no effect was observed for the LT-ΔMUR1 mutant. F: ATOH1-HA construct was transfected in absence or presence of increased amounts of truncated LT from WaGa and AlDo cell lines, the MUR1 region lacking from this latter.

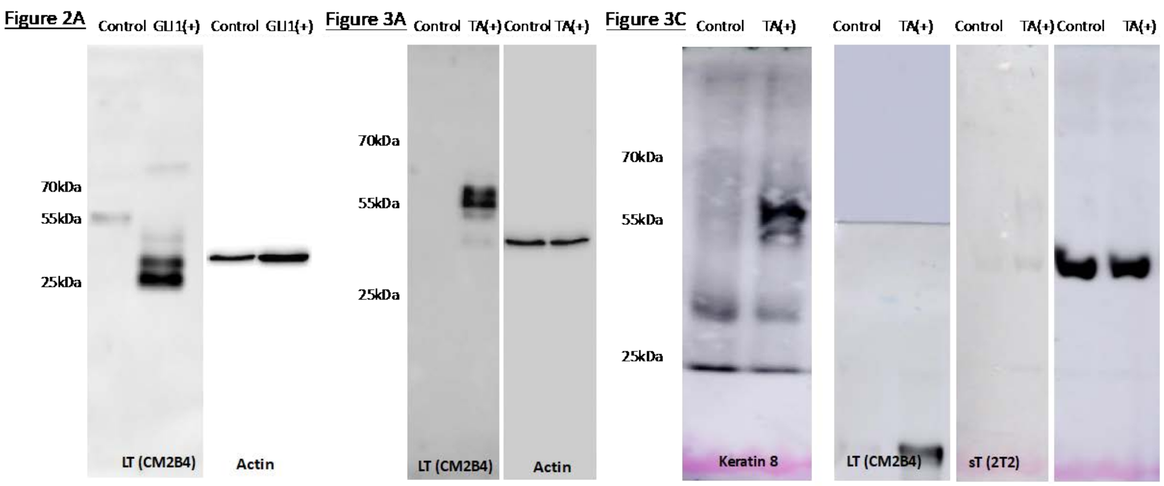


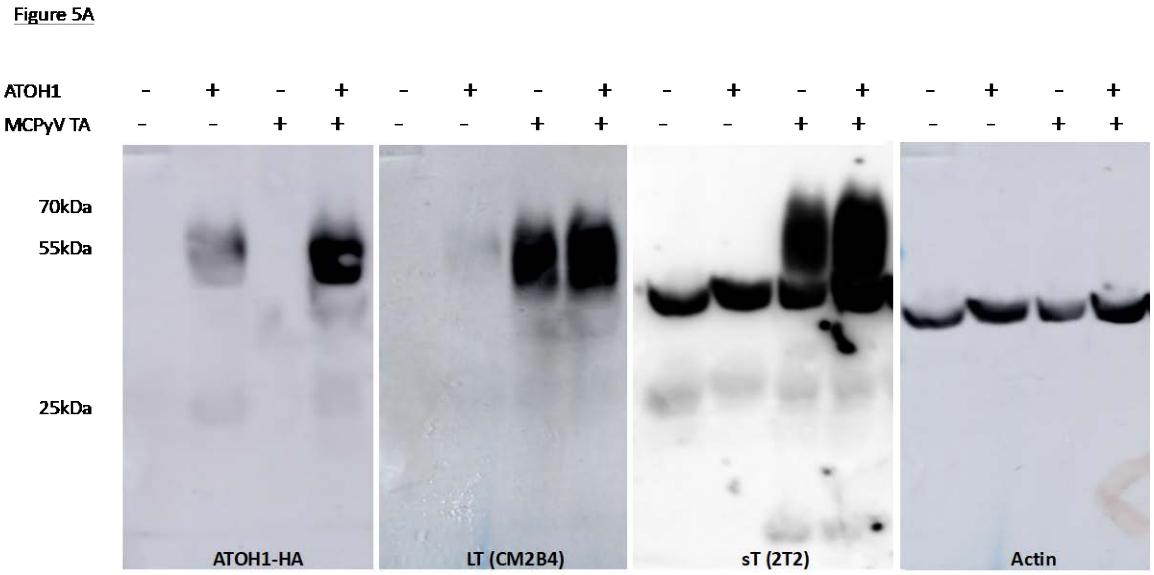


**Figure S8.** Continued.

Figure 5B

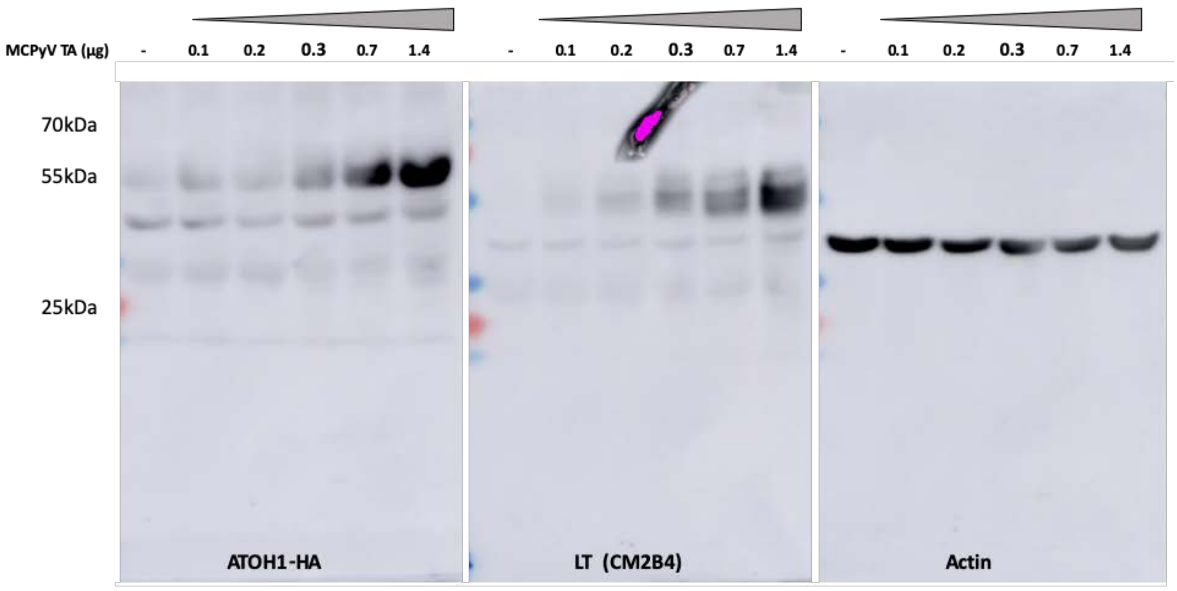


Figure 5C

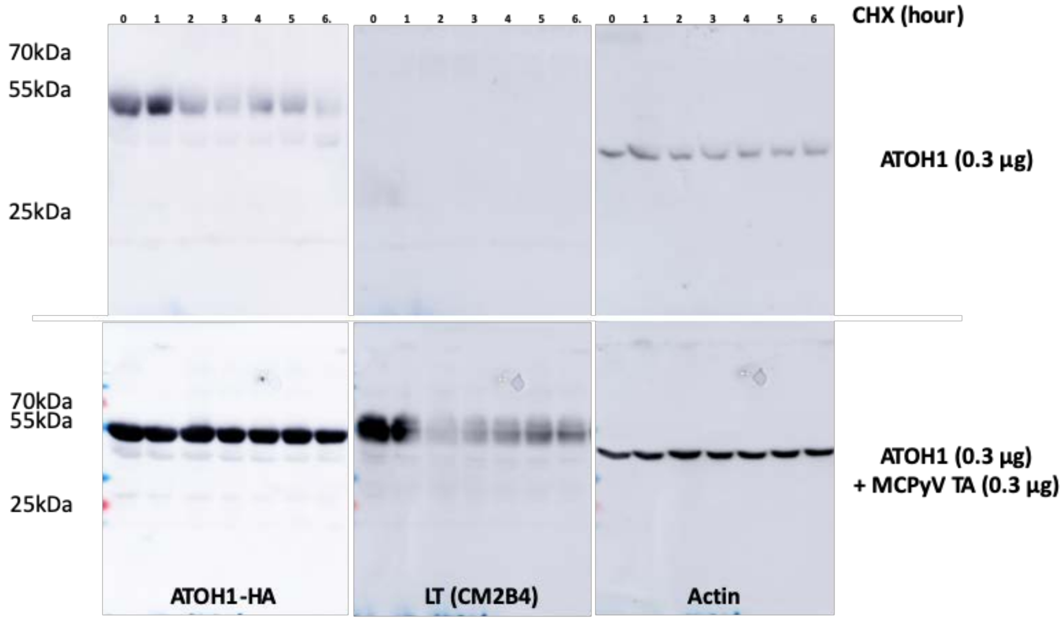


**Figure S8.** Continued.

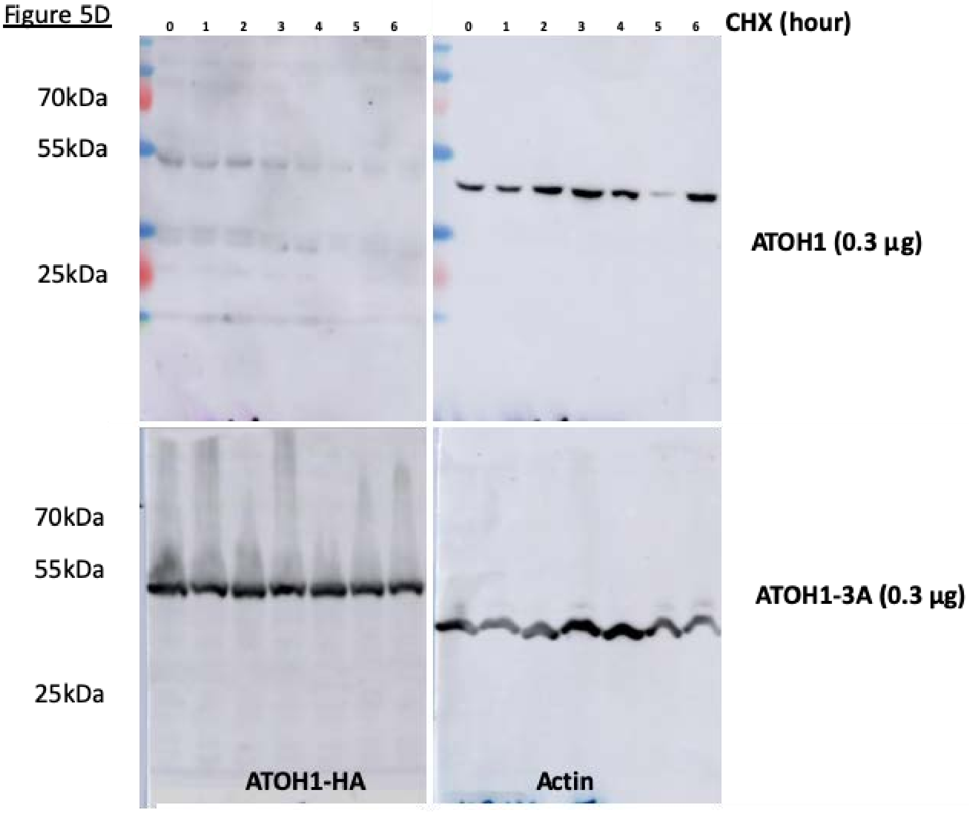


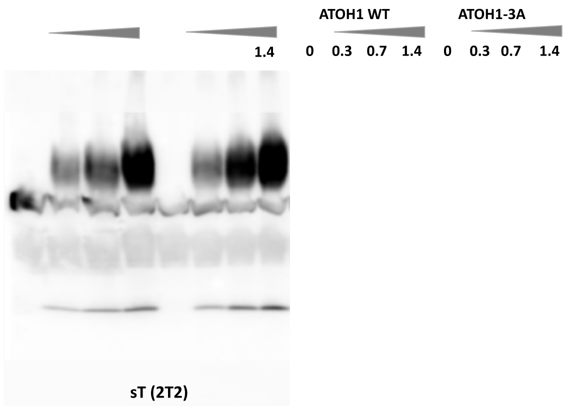


**Figure S8.** Continued.

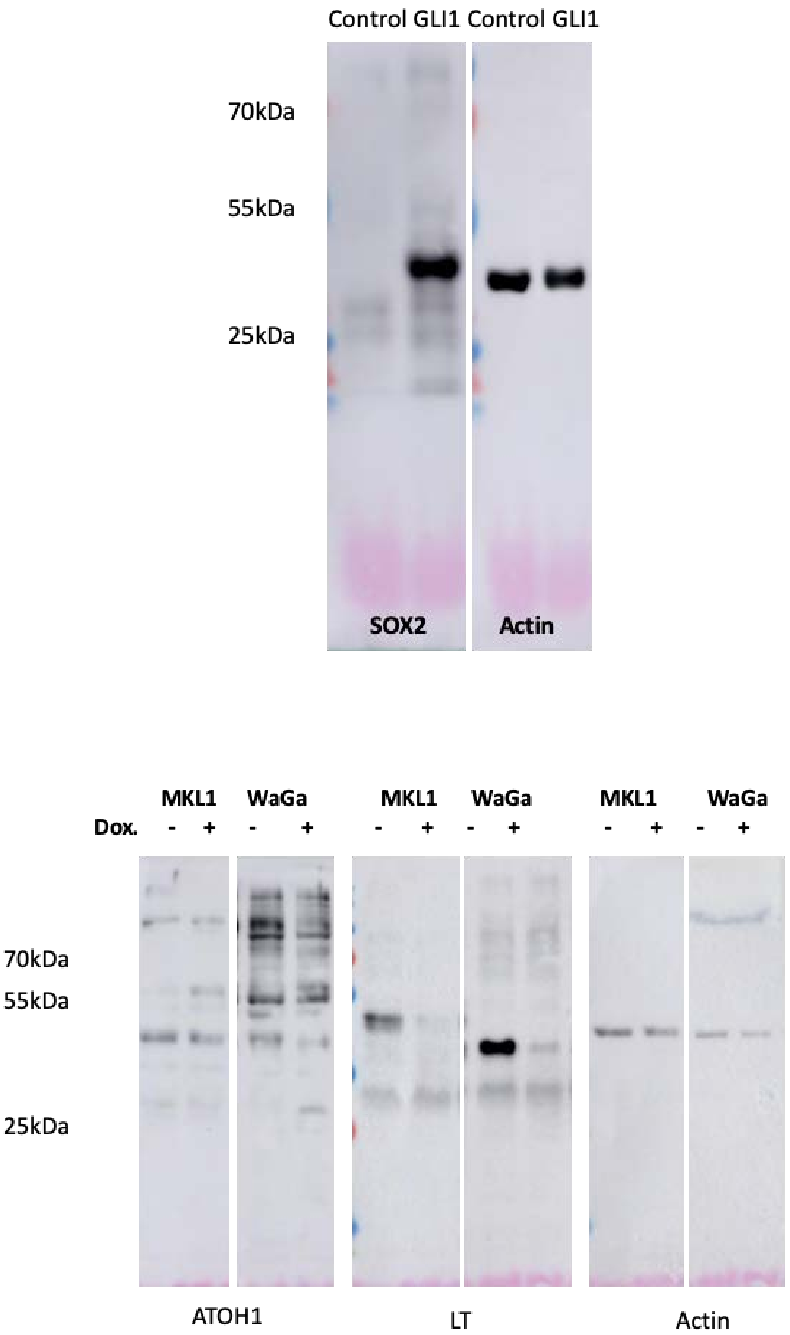


**Figure S8.** Continued.

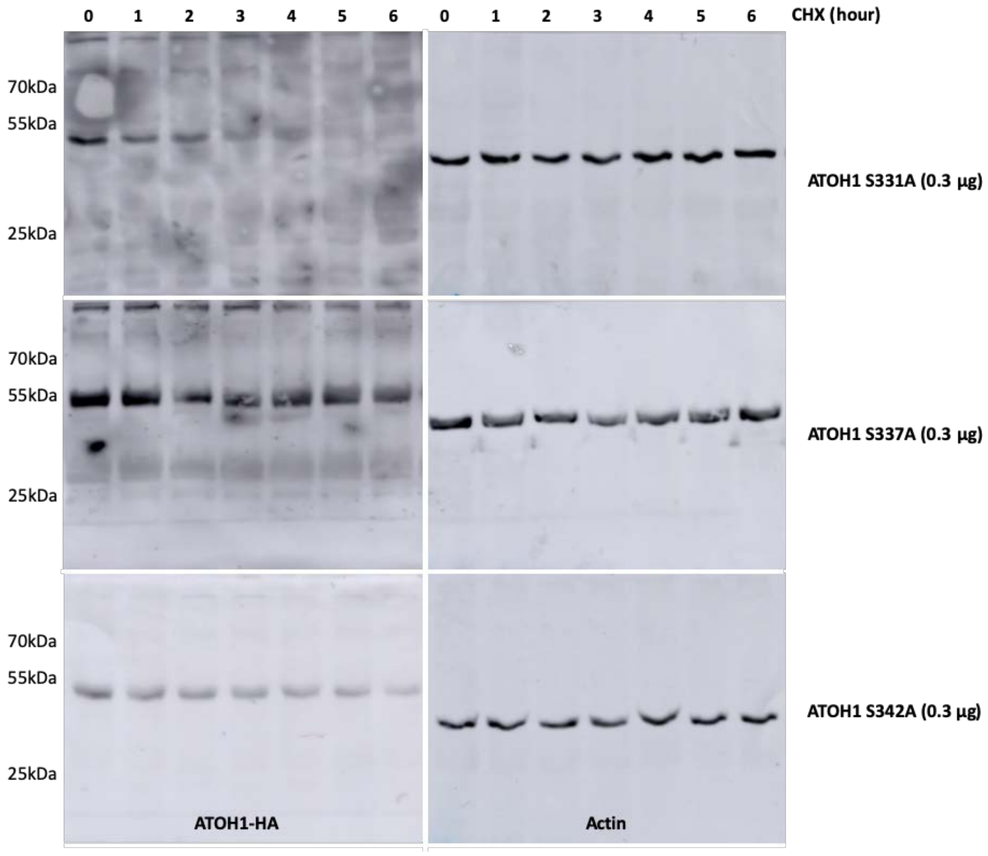


**Figure S8.** Continued.

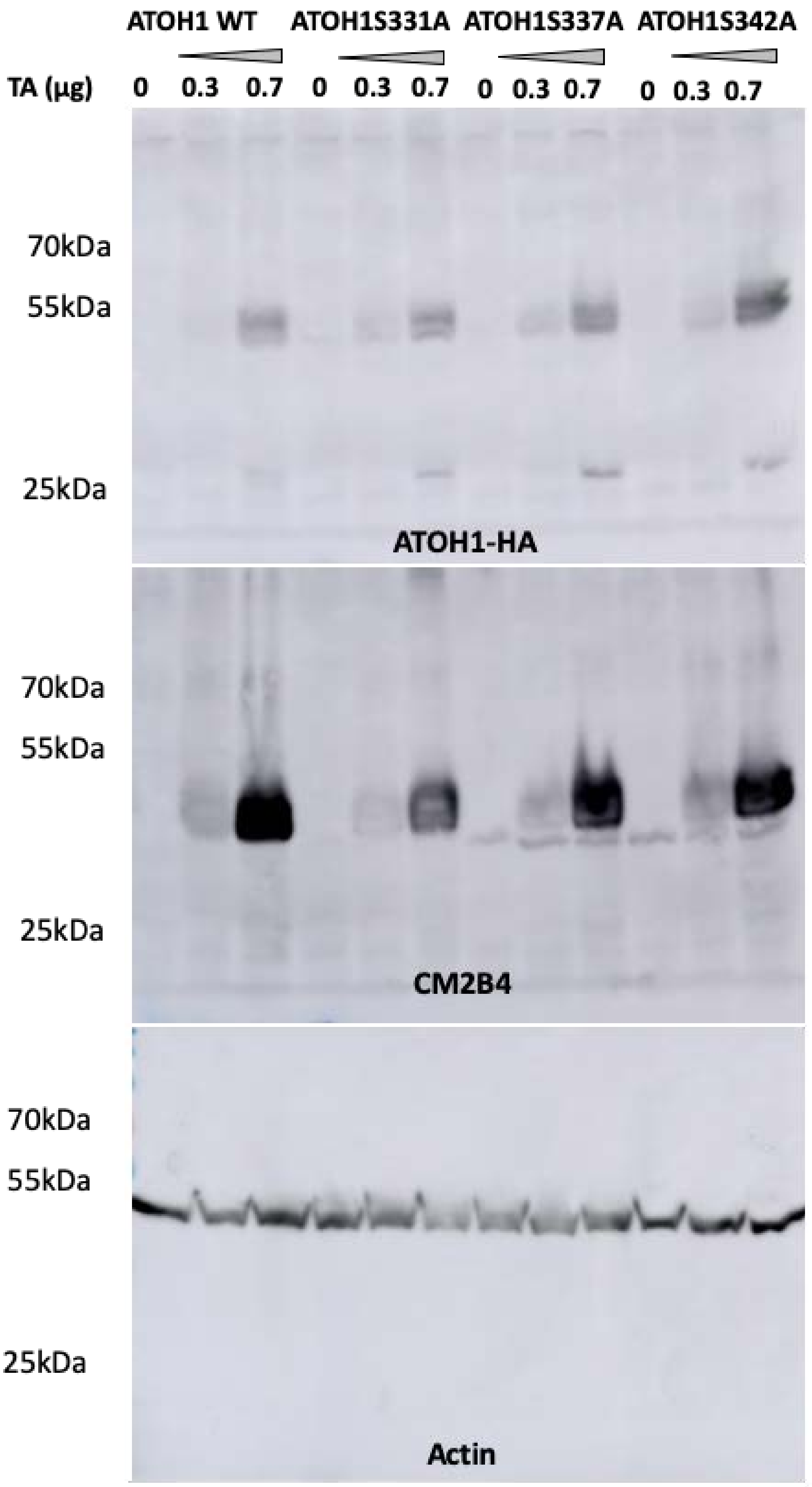


**Figure S8.** Continued.

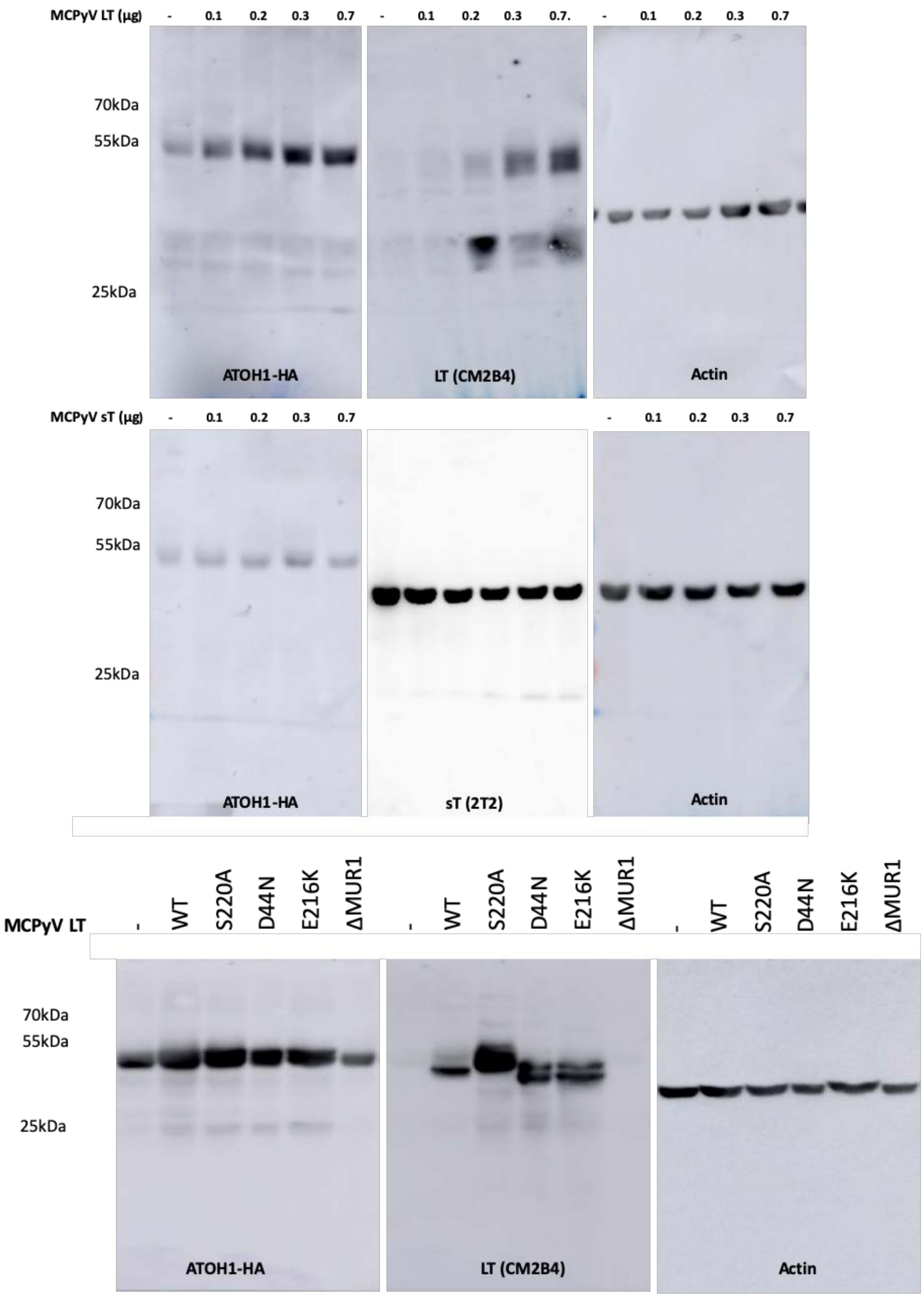


**Figure S8.** Continued.

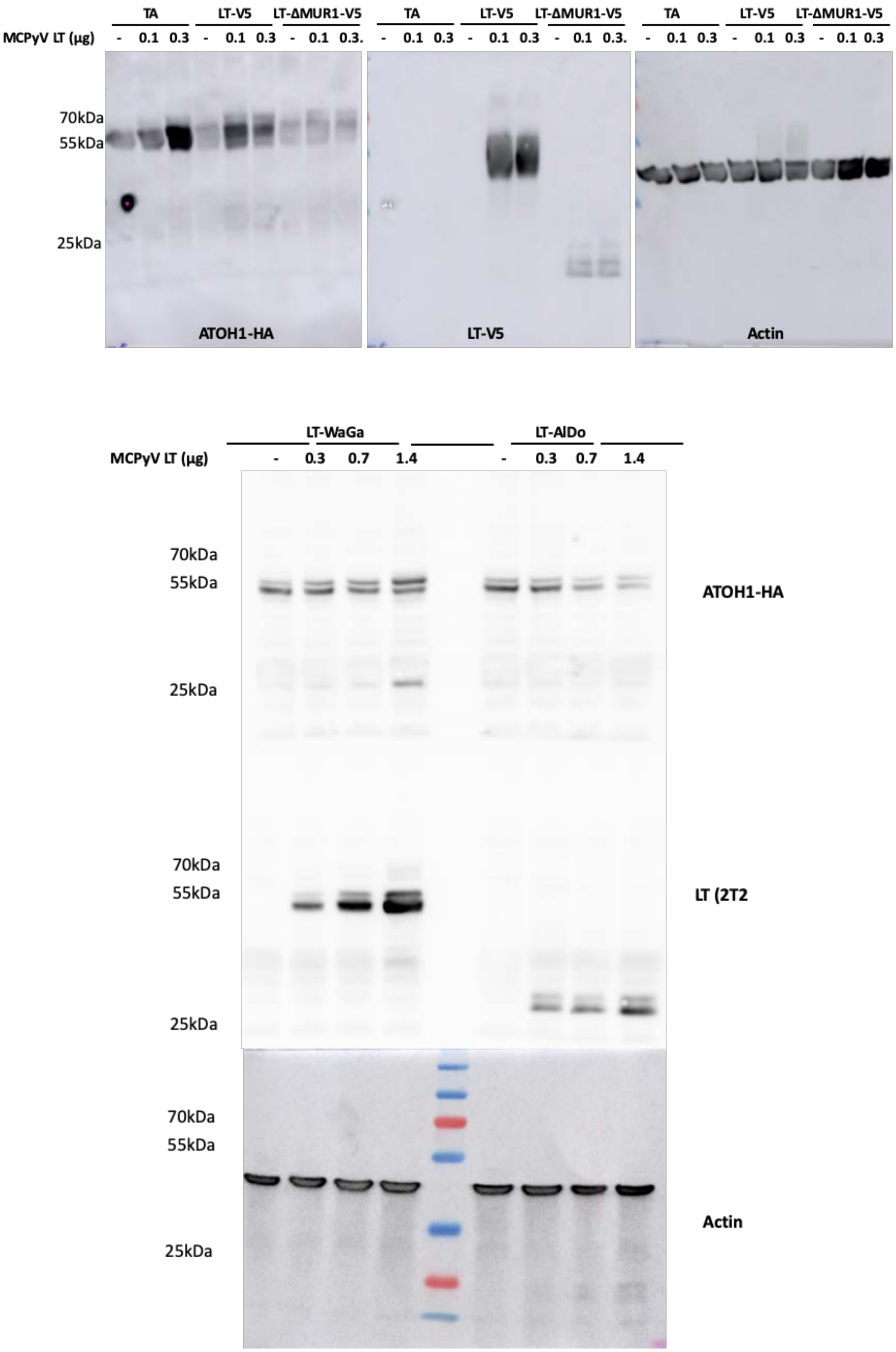


**Figure S8.** Uncropped Western blot membranes primary acquisitions.

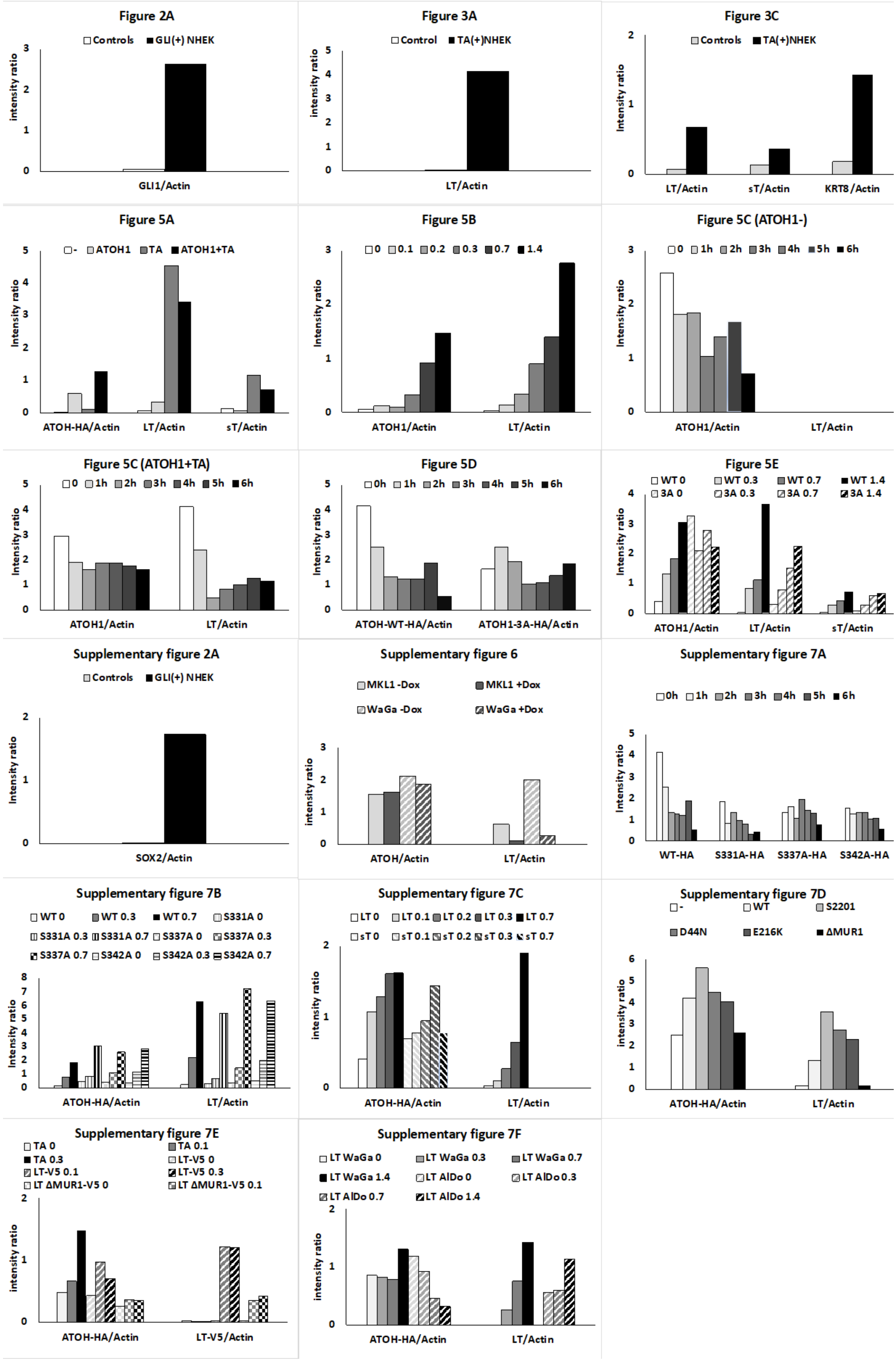


**Figure S9.** Signal quantification of the Western blots.

© 2020 by the authors. Licensee MDPI, Basel, Switzerland. This article is an open access article distributed under the terms and conditions of the Creative Commons Attribution (CC BY) license (http://creativecommons.org/licenses/by/4.0/).
